# Rats show direct reciprocity when interacting with multiple partners

**DOI:** 10.1038/s41598-021-82526-4

**Published:** 2021-02-05

**Authors:** Nina Kettler, Manon K. Schweinfurth, Michael Taborsky

**Affiliations:** 1 Institute of Ecology and Evolution, University of Bern, Wohlenstr. 50a, 3032 Hinterkappelen, Switzerland; 2 School of Psychology and Neuroscience, University of St Andrews,, St Mary’s Quad, St Andrews, KY16 9JP Scotland

**Keywords:** Ecology, Behavioural ecology

## Abstract

Direct reciprocity, where individuals apply the decision rule ‘help someone who has helped you’, is believed to be rare in non-human animals due to its high cognitive demands. Especially if previous encounters with several partners need to be correctly remembered, animals might either stop reciprocating favours previously received from an individual, or switch to the simpler generalized reciprocity mechanism. Here we tested the decision rules Norway rats apply when interacting with multiple partners before being able to return received help. In a sequential prisoner’s dilemma situation, focal subjects encountered four different partners that were either helpful or not, on four consecutive days. On the fifth day, the focal subject was paired with one of the previous four partners and given the opportunity to provide it with food. The focal rats returned received help by closely matching the quantity of help their partner had previously provided, independently of the time delay between received and given help, and independently of the ultimate interaction preceding the test. This shows that direct reciprocity is not limited to dyadic situations in Norway rats, suggesting that cognitive demands involved in applying the required decision rules can be met by non-human animals even when they interact with multiple partners differing in helping propensity.

## Introduction

Altruistic behaviour, where a donor provides benefits to a recipient at a cost to itself, is difficult to explain in evolutionary terms if it occurs between unrelated individuals^1,2^. A mechanism that has been proposed to explain the evolution and maintenance of altruism in a population is reciprocity, where individuals base their decision to cooperate on experienced past interactions and henceforth expected future help^3^. Three behavioural decision rules can establish evolutionarily stable levels of reciprocity in a population. Direct reciprocity reflects the rule ‘help someone who has helped you’^3,4^. A simpler decision rule underlies generalized reciprocity, where individuals ‘help anyone if helped by someone’^5,6^. Indirect reciprocity, in contrast, is based on third party information, where individuals ‘help someone who has helped somebody else’^7,8^. These decision rules involve different cognitive demands, with generalized reciprocity having no need for individual recognition but only the memory of anonymous cooperative experience, and indirect reciprocity at the other end of the scale requiring third party information and specific social recognition.

Reciprocal decisions depend on past interactions and thus require some form of social memory. This is probably the reason why humans that are superior to others in memory tasks also perform better in reciprocity paradigms^9^. As a consequence of these cognitive demands, the more information that needs to be encoded, the more errors are likely to occur in situations requiring decisions about reciprocating previously received help; human subjects asked to recall the behaviour of five, ten or fifteen partners showed error rates between 10 and 24%, which were increasing with the number of partners and intervening events^10^. Such error rates could cause reciprocal cooperation to become unstable in a population^10^. Humans playing the prisoner’s dilemma game performed more generously when a different, memory demanding game was played between subsequent interactions, which interfered with the subjects’ working memory^11^. Again, this suggests that memory requirements can significantly impact decisions to reciprocate received help.

If memory is constrained, individuals may develop simpler strategies to reciprocate help. For instance, humans were shown to remember the most saliently behaving partners in a reciprocity game^12^. By only remembering the few defectors present in a group, memory requirements can thereby be reduced, and reciprocity can be maintained. Furthermore, focusing only on the last move of a partner instead of using information of more than one previous interactions may help to maintain reciprocal cooperation in situations involving enhanced memory challenges^11^. The use of simple decision rules in complex situations has been shown also in other contexts, such as male courtship behaviour in sticklebacks or the choice of migratory routes in birds^13–16^. Given constraints on cognitive processes, selection may cause individuals to not show an optimal behavioural response in every single situation but apply rules that work well on average^16–18^.

In comparison to humans^19–21^, only few studies have yet experimentally scrutinized whether non-human animals use the cognitively less demanding behavioural strategy ‘generalized reciprocity’ when given the chance to reciprocate received help (but see for example: capuchin monkeys^22^, chimpanzees^23^, domestic dogs^24^, Norway rats^25–28^). Hitherto it is not known whether non-human animals switch from applying direct reciprocity rules to the simpler generalized reciprocity decision rule when memorizing the previous behaviour of individual partners becomes challenging. Therefore, here we asked whether female Norway rats (*Rattus norvegicus*) continue using direct reciprocity rules when experiencing several partners successively that either provided or withheld help in the form of food donations, or whether they would switch to applying the simpler generalized reciprocity rules in such situations. Direct reciprocity can lead to higher cooperation levels in female Norway rats than generalized reciprocity ^26^.

Norway rats have been experimentally shown to reciprocate food donations and allogrooming in variants of the prisoner’s dilemma paradigm using both direct and generalized reciprocity rules^25–35^, and to trade different tasks and commodities among each other in a reciprocal manner^32,33^. In nature they are known to share food and huddle together for thermoregulation^36^.

In this study, we asked whether wild-type Norway rats are able to apply direct reciprocity decision rules in a complex social setting. Focal rats experienced four different social partners over four consecutive days. On the fifth day they could provide food to an individual that was randomly chosen from these four previous partners. In the first treatment, all partners were cooperating (i.e. providing food) except for the last one, which was defecting (i.e. did not provide food). In the second treatment, all partners were defecting except for the last one, which was cooperating. If rats used more simple decision rules when memorizing specific information is challenging, they would be expected to cooperate according to their last experience and apply generalized reciprocity rules. In contrast, we predicted that rats memorize the cooperation level of the four previously unfamiliar partners, the behaviour of which they had experienced only once during a 7 min encounter several days before. Hence their decision would not be determined by their last interaction but by the specific previous help they received from the partner they met during the test, thereby applying direct reciprocity rules.

## Methods

### Experimental subjects and housing conditions

49 adult female Norway rats (*Rattus norvegicus*; source: Animal Physiology Department, University of Groningen, Netherlands) were used in this experiment. They were housed in cages (80 × 50 × 37.5 cm) of three to five same-sex littermates and were ear punched to identify them. The rats were habituated to handling prior to testing and hence showed no signs of stress when being taken out of the cage and transported to the experimental room. To avoid social interaction between the housing groups, the cages were separated from each other through opaque dividers. The temperature in the room was kept constant at 21 °C ± 2 °C, with a relative humidity of 55–65% and an inversed 12/12 h light/dark cycle with lights out at 8:30 a.m. and 30 min of dusk and dawn. All interactions with the rats were conducted during their dark phase, as rats are primarily nocturnal^37^.

### Experimental setup and pulling apparatus

The experimental setup was based on a pulling task designed for food provisioning of one individual to another, where the donor did not receive a reward by its action^25,26^. The experimental cage (80 × 50 × 37.5 cm) was divided by a wire mesh to create two compartments, allowing full sensory contact between the focal rat and its partner. A movable tray that was connected to a stick was placed in front of the cage. By pulling the stick, one rat could move the tray towards the experimental cage and provide an oat flake as food reward to the rat in the adjacent compartment. Subsequently the tray was reloaded by a food dispenser (Fig. S1; cf. ^27^), which was remotely controlled by the experimenter.

### Pre-experimental training

Before testing, the individuals were first trained to operate the apparatus in a solo pulling phase, after which they experienced a social training phase. The solo training consisted of six sessions per individual. All rats reached the learning criterion to pull the tray for themselves at least 50 times within 10 min. Next, individuals were paired with another rat from their cage (‘training partner’) for the entire social training. Now pulling the stick resulted in a food reward for their partner only. In the subsequent trial, the partner could pull the stick and provide food for the other rat in return. Over the course of fourteen sessions, we gradually increased the intervals between switching the donor and recipient roles up to a 24-h break in between (see Table S2). In the beginning of the social training, every individual pulled once before the roles were exchanged (sessions one and two). Afterwards, they pulled twice (sessions three and four) and thereafter four times (sessions five and six) before the roles were switched. Every session lasted 14 min. Starting with session seven, we allowed each individual to pull for a certain amount of time, independently of how many times it pulled during that time. In sessions seven and eight, one individual was allowed to pull for two times 4 min and the other for 4 min in between (12 min total). From session nine onward, every individual could provide food to its training partner for 7 min, which corresponds to the later experimental period, after which we switched the roles. Within training dyads, we counterbalanced which partner started the training session and in which compartment they were placed. No rat experienced more than one training session per day.

### Reciprocity test

We allocated our rats to three groups based on their pulling frequency during the last six sessions of the social training phase. The individuals with the highest pulling frequencies (average pulling frequencies within 7 min over 6 training sessions: min. 8; max. 10.83) were used as providers of cooperative experience (‘cooperators’; *N* = 12), whereas the ones with the lowest pulling frequencies (average pulling frequencies within 7 min over 6 training sessions: min. 0.5; max. 3.83) were used as providers of non-cooperative experience (‘defectors’; *N* = 13). The remaining individuals (average pulling frequencies within 7 min over 6 training sessions: min. 4; max. 7.33) were used as test subjects (‘focal rats’; *N* = 24).

**Figure 1:**
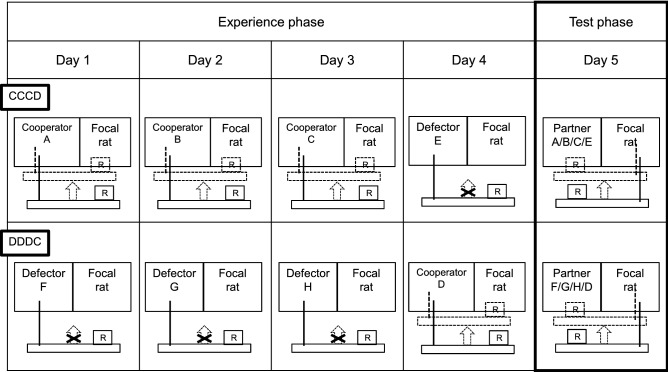
Experimental setup. During the experience phase, focal rats were paired with cooperating partners that provided food rewards (R) to them, or defecting partners that did not provide food rewards to them. In one treatment (CCCD), focal rats were paired with three different cooperators on three consecutive days, and a defector on the fourth day. In another treatment (DDDC), focal rats were paired with three different defectors on three consecutive days, and a cooperator on the fourth day. During the test phase, focal rats could provide food rewards to one of the partners they had encountered on the previous four days (partner A, B, C or E in treatment CCCD, and partner F, G, H or D in treatment DDDC).

The test consisted of an experience phase and a test phase, similar to Schweinfurth and Taborksy^38^. During the experience phase, focal rats were paired with four different partners on four consecutive days (Fig. 1). In treatment CCCD, the first three partners were all cooperators, while the last partner was a defector. In treatment DDDC, the first three partners were all defectors, while the last partner was a cooperator. For the test phase on day five, focal rats were paired with one of their previous partners for which they now could provide food. To ensure that ‘defectors’ could not provide food to the focal rats during the experience phase, the platform was fixed by blocking the switch that allowed the tray to be moved toward the cage. This switch was never blocked during the test phase.

Each focal received both treatments with a 15-days break in between to prevent carryover effects. The partners in the test phase were chosen in a pseudorandomized manner and the sequence was kept constant over the two treatments regarding the day on which they had previously encountered their respective test partner. This ensured that each focal rat would be allowed to provide food to both a cooperator and a defector during the experiment after the same time interval (e.g., in the test phase on day five, ‘focal rat 1′ was allowed to provide food to the cooperator experienced on day two in treatment CCCD, and correspondingly to the defector experienced on day two in treatment DDDC). Every trial during the experience and test phases lasted for 7 min, with 1 min of acclimatisation in the test cage prior to the start of the experiment. All rats used in the tests were unfamiliar and unrelated to each other. In addition, no test subject experienced more than one trial per day. The order of focal rats was randomized and kept constant during the test, meaning they were always tested at similar times of day, which reduced the possibility of behavioural fluctuations due to the state of the individual. In addition, we pseudorandomized the treatment order to ensure that all treatments were tested equally often in every testing week.

### Statistical analyses

All graphs and statistical analyses were performed using R (version 1.0.136, with R Studio, packages: ggplot2, FSA, lme4, survival).

The numbers of food rewards the focal and partner rats provided in both the experience and test phases were recorded. They were not normally distributed (Shapiro–Wilk normality test: W = 0.92, *p* = 0.004). First, we analysed whether focal rats that experienced several partners would base their decision to donate food to a partner in the test phase on this particular partner’s helping propensity during the experience phase (direct reciprocity), or whether they would instead consider the experience during their last encounter, which involved a different individual (generalized reciprocity). For this, we used a generalized linear mixed model. We included the focal rats’ number of provided food rewards as a response variable. In addition, we included the role of the partner (i.e. cooperating or defecting) as a fixed factor, the day of encounter with their partner in the experience phase (i.e. day one, two, three or four) as a continuous variable, and the treatment (i.e. CCCD or DDDC) as a fixed factor. The ‘treatment factor’ was used to determine whether the focal rats based the number of food rewards they provided on their last encounter with any individual, or their last encounter with a specific individual. Furthermore, we corrected for using the same individuals repeatedly by including the focal and the partner rat’s identity as a random effect. We assumed a Poisson distribution of the number of food rewards provided and tested for model fit, which was overall good. We checked for an interaction effect between the role of the partner and the day of encounter with the partner and found none (GLMM: β ± SE = − 0.31 ± 0.21, *Z* = − 1.50, *N* = 47, *p* = 0.14). The interaction was thus dropped from the model^39^.

Second, we analysed whether the number of food rewards provided by focal rats was correlated with the number of food rewards they had received from their cooperating partner by using a Spearman rank correlation test. Third, we used a two-tailed Wilcoxon matched-pairs signed-ranks test to determine whether the latencies to the first food reward provided by the focal individuals would significantly vary with the previous helpfulness of the partner.

### Ethical note

In accordance with animal welfare legislation of Switzerland (Tierschutzverordnung Schweiz 04/2008) we provided the rats with enriched cages (80 × 50 × 37.5 cm) containing various materials, i.e., a wooden house and board, a channel, a piece of wood to nibble, an empty toilet roll to play with, digging material (wood shavings), nest-building material (hay) and a salt block. Food (conventional rat pellets) and water were provided ad libitum. In addition, we provided rats every day with a corn mix or with fresh fruits and vegetables. The housing of the animals and the experimental procedure were authorized by the Swiss Federal Veterinary Office under license BE 55/18. No injuries occurred during our experiments. This study conformed to the ARRIVE guidelines^40^.

## Results

**Figure 2:**
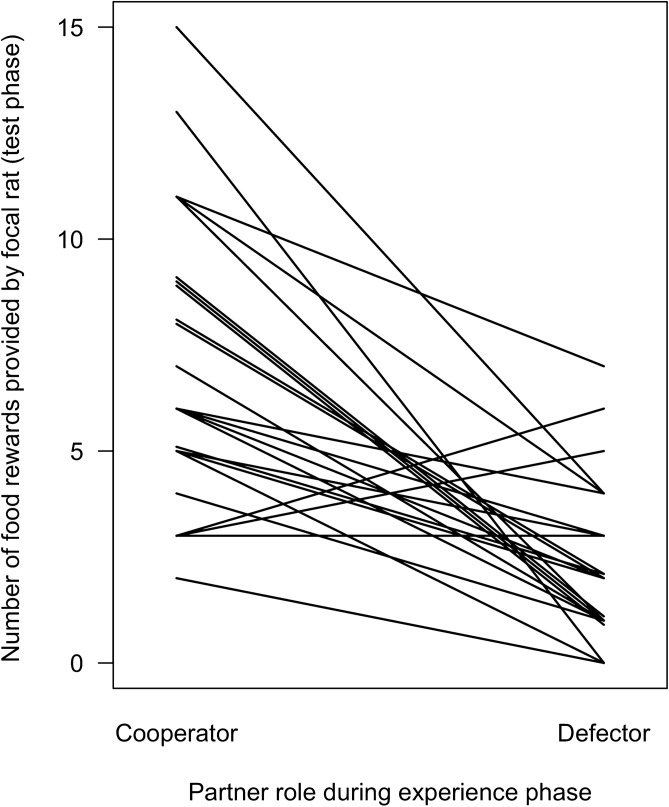
Total number of food rewards provided by focal rats to their cooperating and defecting partners. 21 out of 24 focal individuals provided food rewards more often to the previously cooperating than to the previously defecting partner. Every line represents the number of food rewards provided by one focal individual to its respective previous cooperator (left) and defector (right).

**Figure 3:**
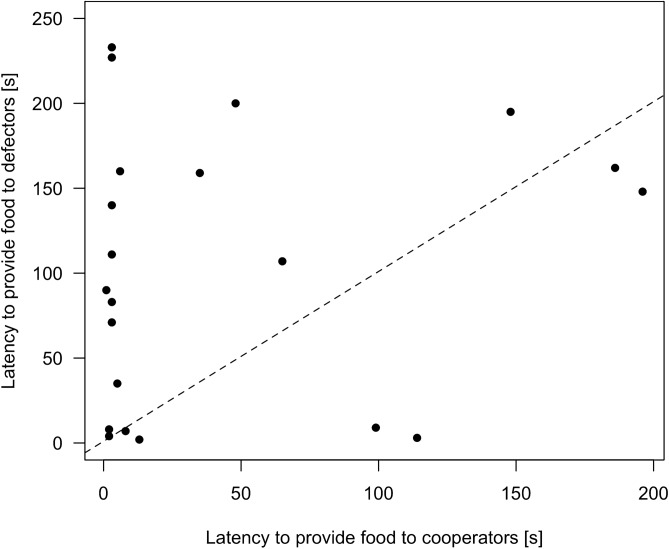
Latencies to first food pulling of focal rats for their previously cooperating partner *vs*. for their previously defecting partner. The previous helpfulness of the partner influenced the latency (in seconds) of test subjects to provide the first food reward to its partner. Every data point represents the focal individual’s latency to the first food provisioning of the cooperating partner compared with that of the defecting partner. Data points above the dashed line represent focal individuals who pulled earlier for the cooperating partner than for the defecting partner. This is inversed for data points below the dashed line. Significantly more focal individuals pulled earlier for their cooperating partner than for the defecting partner.

**Figure 4:**
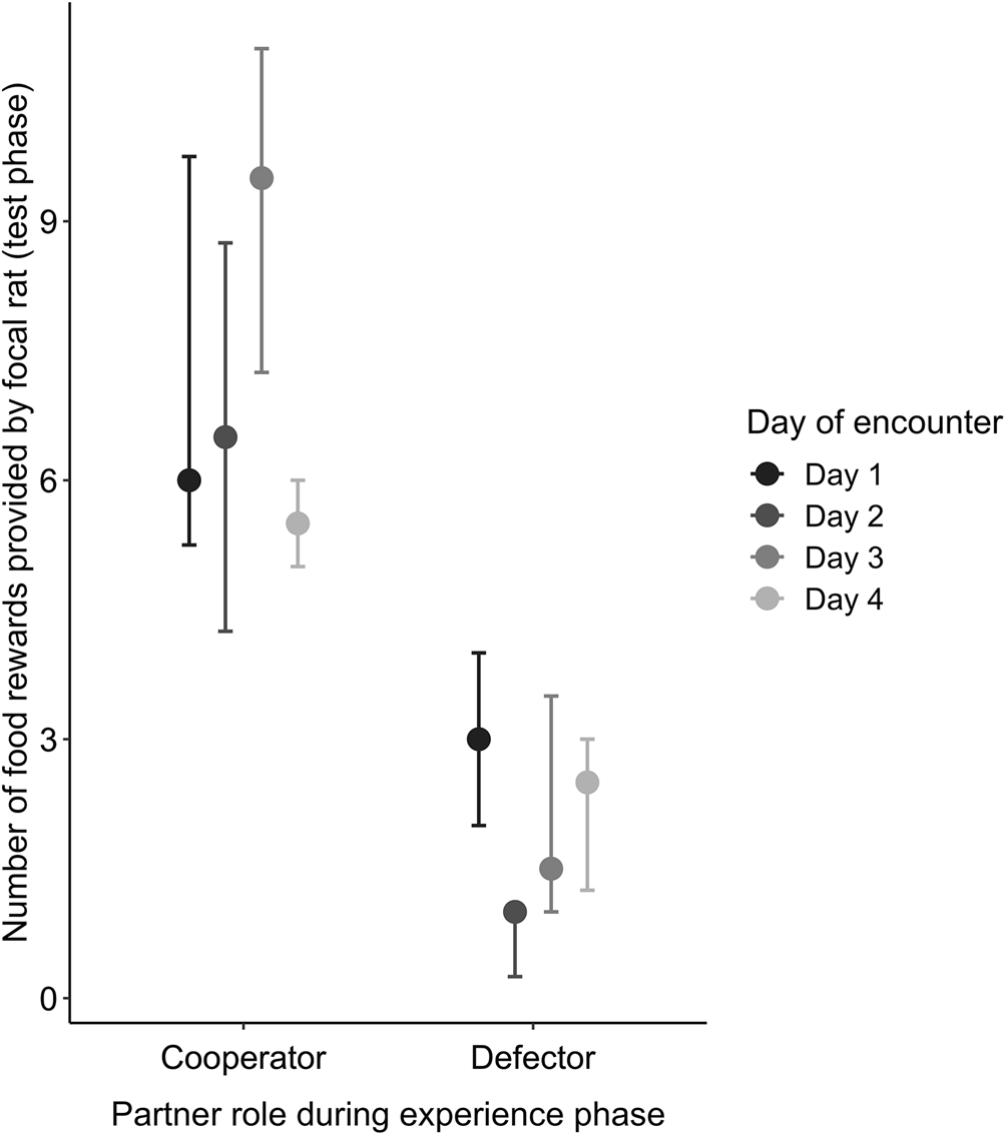
Total number of food rewards provided by focal rats to their cooperating and defecting partners in relation to the point in time when they had experienced their partners. Focal rats provided food rewards more often to previously cooperating than to previously defecting partners, which did not differ between partners experienced a longer or shorter while ago. Medians and interquartile ranges are shown of the focal rats’ pulling frequencies for their partners that had been experienced either on days one, two, three or four (each *N* = 6).

Focal rats provided more food rewards to cooperators than to defectors (GLMM: β ± SE = -1.01 ± 0.18, *Z* = − 5.73, *N* = 47, *p* < 0.001; Fig. 2), and they pulled significantly earlier for cooperators than for defectors (Wilcoxon test, n = 24, V = 47, *p* = 0.016; median cooperators: 7 s ﻿[range 1–196]; median defectors: 107 s ﻿[range 2–233 or not pulling at all]; Fig. 3). The number of food rewards provided was neither influenced by their last helping experience (GLMM; β ± SE = − 0.27 ± 0.17, *Z* = − 1.54, *N* = 47, *p* = 0.12; Fig. 4), nor by the position in the sequence of partner rats in which they had been encountered during the experience phase (GLMM: β ± SE = − 0.001 ± 0.078, *Z* = − 0.02, *N* = 47, *p* = 0.99; Fig. 4).

**Figure 5:**
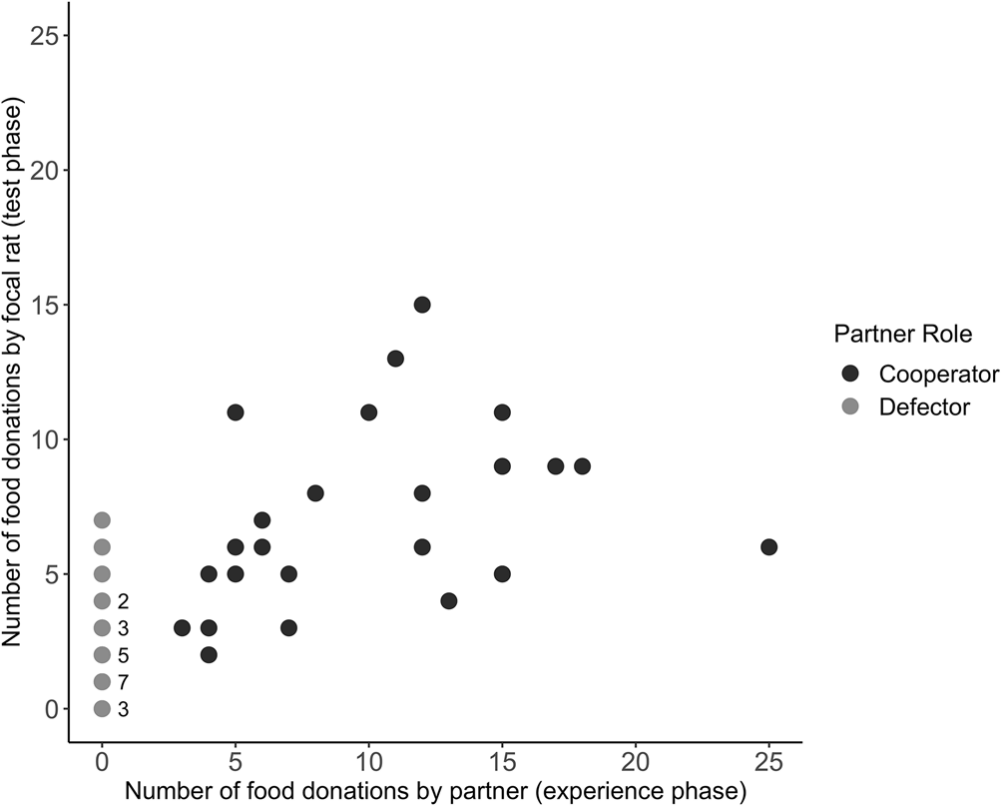
Total number of food rewards provided by focal rats to specific partners in relation to the number of food rewards previously received from them. Food donations exchanged between the focal individuals and their partners were positively correlated. For the statistical analysis, only the data from “cooperators” were considered. Numbers beside points denote the number of overlapping data points. Focal individuals donated more food to partners that had provided more food to them during the experience phase than to those that had been less generous.

The focal rats’ pulling frequency during the test phase was positively correlated with the pulling frequency of their cooperative partners during the experience phase (Spearman rank correlation: S = 1172.5, r = 0.49, *N* = 47, *p* < 0.015; Fig. 5). The median difference between the number of food rewards focal rats provided to previous cooperators and the number of food rewards they had previously received from these cooperators was -1.5 (interquartile range − 6 to + 1), i.e. the focal rats provided the cooperators on average with slightly less food rewards than they had previously received from them.

## Discussion

Our results show that rats apply direct reciprocity rules when interacting with four different partners over a period of five days. In this situation, rather than switching to the simpler decision rule that characterizes generalized reciprocity, the focal rats provided food rewards based on the amount of previously received food rewards from a specific partner, even if this had happened four days ago. They also provided food rewards earlier to a cooperative partner than to a defecting partner. This reveals that Norway rats are able to memorise the cooperative behaviour of previous partners over several days after interacting with others of varying cooperativeness in between.

There is evidence that rats can somewhat economize on memory demands if interacting repeatedly with multiple partners, which might enable them to use direct reciprocity even under challenging conditions, such as when interacting with multiple interaction partners like in our experiment. Rats apparently do not form social bonds^41^, and they do not consider or integrate several successive experiences with a social partner^38^. Hence, they may reduce the memory requirements for reciprocal cooperation by discarding information about earlier behaviour of their partner and regularly updating old with new information. Rather than switching to the simpler generalized reciprocity decision rule when the number of partners and intervening events increases, rats apparently keep following the more efficient and cheat-proof direct reciprocity rules. They may hence use generalized reciprocity only if information on a partner’s helping propensity is lacking, which is in accordance with the ‘hierarchical information hypothesis’ of reciprocal cooperation^26^. Under natural conditions, rats sometimes live in groups of more than 150 individuals^42,43^. In such situations it is likely that rats will encounter partners of which they do not know or exactly remember the last interaction valence; in primates, for example, the ability to monitor social encounters with different partners is apparently dropping with increasing interaction numbers^44^, which is most likely due to rising cognitive demands.

Interestingly, rats distinguished between cooperating and defecting partners equally well when these had been experienced between one and four days before the test. Apparently, rats may use their episodic-like memory^49^ also in the social context by remembering who did what to them, even if an interaction had happened several days before^38^, which seems to be in agreement with results obtained both in laboratory rats^45^ and other animals in non-social tasks (reviewed in ^46^). This ability allows rats to apply a tit-for-tat like strategy (i.e., respond to the last interaction with a social partner by returning like for like) as found in a previous study^38^ even if they had interacted with other social partners in between their last interaction with the current partner. This is advantageous especially in animals like Norway rats that may live in large groups and hence interact with many different individuals ^42,47,48^, because it enables reciprocal cooperation also under such complex conditions. Decision rules making use of only the last encounters with a particular partner can evolve more easily, which is why tit-for-tat like strategies are so powerful in theoretical models^4^.

Remarkably, the rats responded quite accurately also to the quantity of food rewards received from their previous partner, as received and provided food rewards were positively correlated between cooperators and focal rats. This adds to the accumulating evidence for enhanced cognitive and social capabilities of rodents^31,49,50^.

Future research will show how widespread the ability shown by Norway rats is among animals to cope with a socially challenging situation by successfully maintaining direct reciprocity. The frequent occurrence of reciprocal cooperation in animals^2^ may suggest that indeed, many social species might be similarly competent in memorizing the information required for using direct reciprocity rules in complex social settings. This is corroborated, for instance, by the observation that ravens distinguish between human experimenters diverging in provisioning levels after intervals of at least 1 month, which may suggest that they also possess the memory capacity required for reciprocal cooperation, even if this was not yet tested among conspecifics^51^.

In conclusion, our results show that a cognitively demanding mechanism like direct reciprocity can be maintained by non-human animals in a complex social situation, contrary to common belief^10,12,52^. Rats apparently possess the capacity required to remember the identities and helping propensities of at least four partners over at least four days. They distinguish both qualitative and quantitative information about the helping propensities of those partners and apply direct reciprocity decision rules when returning previously received help to specific social partners. Our setup used a sequential iterated prisoner’s dilemma game with multiple partners, which models the social complexity found in nature more closely than a game confined to two partners as had been previously employed. The results hence demonstrate that Norway rats apply direct reciprocity rules when deciding to help conspecifics under challenging social conditions. This suggests that reciprocal cooperation in non-human animals might not be strongly limited by cognitive constraints, which may explain why reciprocity and the exchange of help seem to be widespread in animals^2^.

## Supplementary Information

Supplementary Information 1.

Supplementary Information 2.

Supplementary Information 3.
