# Supplementary figures and images for: Rats show direct reciprocity when interacting with multiple partners
Source: Sci Rep. 2021 Feb 5;11:3228. doi: 10.1038/s41598-021-82526-4 (PMC7864983; doi:10.1038/s41598-021-82526-4)

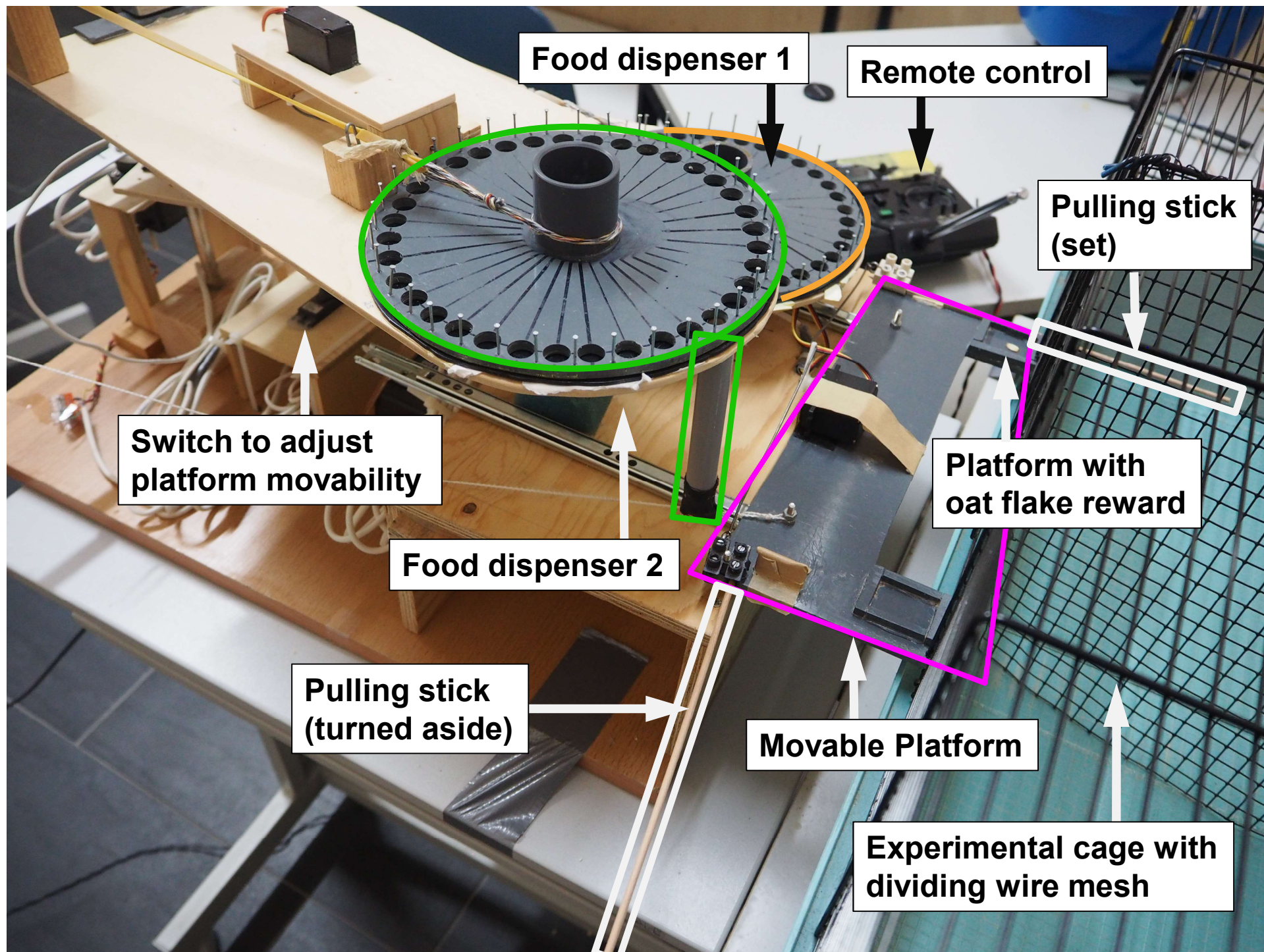

Supplement: Supplementary file 1 — Supplementary Information 1. [file 41598_2021_82526_MOESM1_ESM.pdf]
