# Supplementary material for: Rats show direct reciprocity when interacting with multiple partners
Source: Sci Rep. 2021 Feb 5;11:3228. doi: 10.1038/s41598-021-82526-4 (PMC7864983; doi:10.1038/s41598-021-82526-4)
Supplement: Supplementary file 2 — Supplementary Information 2. [file 41598_2021_82526_MOESM2_ESM.docx]

**Rats show direct reciprocity when interacting with multiple partners**

Nina Kettler^1^*, Manon K. Schweinfurth^1,2^ & Michael Taborsky^1^

^1^Institute of Ecology and Evolution, University of Bern, Wohlenstr. 50a, 3032 Hinterkappelen, Switzerland

^2^ School of Psychology & Neuroscience, University of St Andrews, St Mary’s Quad, KY16 9JP St Andrews, Scotland

*Correspondence: nina.kettler@iee.unibe.ch

**Appendix**

**
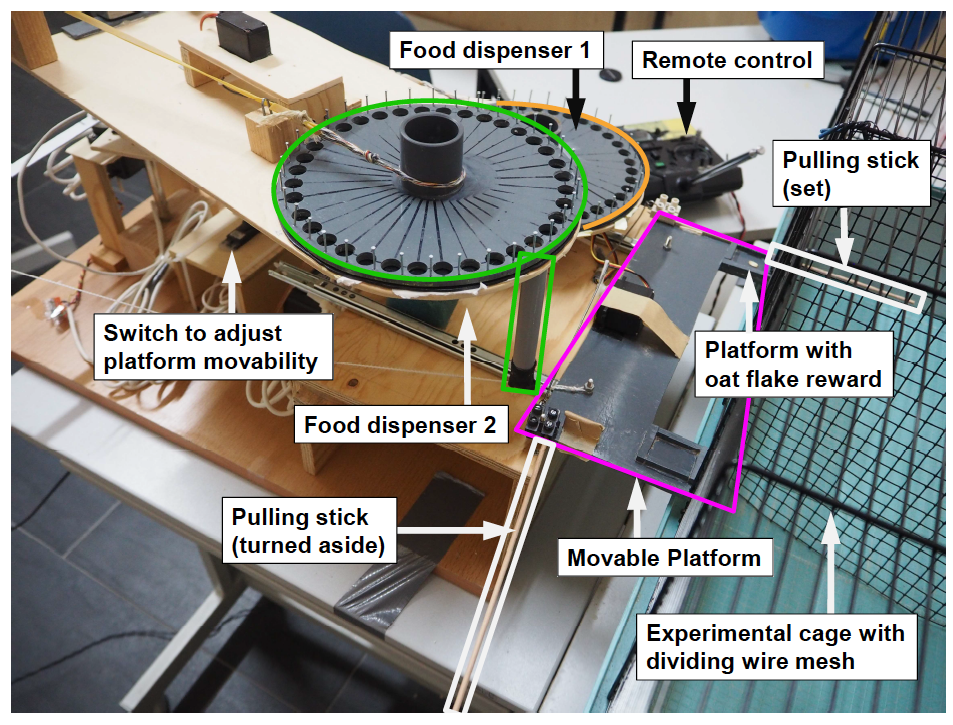
**

**Fig. S1: The pulling apparatus**

By using the pulling apparatus rats could provide a food reward (one oat flake) to their partner by pulling the platform toward the experimental cage if the corresponding stick was set, i.e. if it was reaching inside their compartment. After they pulled the platform into reach of their partner by which the partner received the food reward, the platform was retracted and reloaded by a remote mechanism controlled by the experimenter. The experimenter could also block the movement of the platform by using the switch. This pulling apparatus was developed and implemented by Res Schmid (cf. ^50^).

**Table S2: The social training scheme**

| **Session** | **Individual A** | **Individual B** | **Total time of session** |
| --- | --- | --- | --- |
| **1** | 1 pull ^LF^ | 1 pull | 14 minutes |
| **2** | 1 pulls ^L^ | 1 pulls ^F^ | 14 minutes |
| **3** | 2 pulls ^F^ | 2 pulls ^L^ | 14 minutes |
| **4** | 2 pulls | 2 pulls ^LF^ | 14 minutes |
| **5** | 4 pulls ^LF^ | 4 pulls | 14 minutes |
| **6** | 4 pulls ^L^ | 4 pulls ^F^ | 14 minutes |
| **7** | 2 x 4 min pulling ^F^ | 4 min pulling ^L^ | 12 minutes |
| **8** | 4 min pulling | 2 x 4 min pulling ^LF^ | 12 minutes |
| **9** | 7 min pulling ^LF^ | 7 min pulling | 14 minutes |
| **10** | 7 min pulling ^L^ | 7 min pulling ^F^ | 14 minutes |
| **11** | 7 min pulling ^F^ | 7 min pulling ^L^ | 14 minutes |
| **12** | 7 min pulling | 7 min pulling ^LF^ | 14 minutes |
| **13** | 7 min pulling ^LF^ | 7 min pulling | 7 min 🡪 7 min (after 24h) |
| **14** | 7 min pulling ^L^ | 7 min pulling ^F^ | 7 min 🡪 7 min (after 24h) |

Two individuals were paired to learn how to operate a food-donation paradigm that would provide a food reward (one oat flake) to their respective training partner. The roles of donor and receiver were exchanged at increasing time intervals: In session one, individual A was put in compartment one (^L^; left) and pulled first (^F^), while individual B was in compartment two (right) and pulled second. After A had pulled once and B had eaten the provided food reward, the roles were exchanged. Now B pulled for A once before the roles were again exchanged. The session lasted fourteen minutes. In session two, B started the training session. In session three, the rats’ positions in the compartments were exchanged so that B was in compartment one. A pulled first in session three whereas B pulled first in session four. In both of these sessions each rat pulled twice before the roles were exchanged. Sessions five and six were identical to sessions one and two, respectively, in identity and position of the first puller, but the rats could pull four times for their partner before the roles were exchanged. Starting with session seven, the rats could pull for their partner for a given amount of time instead of a fixed amount of pulls. In session seven, A was allowed to pull for four minutes first, then B, and then A again. This was inverted for session eight. From session nine onwards every individual pulled for seven minutes before the roles were exchanged. Lastly, in sessions thirteen and fourteen, instead of the previous immediate role exchange, the first puller now had to wait 24 hours before their partner was given the opportunity to provide food in return (during which period the rats were transferred back to the holding cage).
